# Motion-induced blindness continues outside visual awareness and without attention

**DOI:** 10.1038/srep11841

**Published:** 2015-07-03

**Authors:** Kevin C. Dieter, Duje Tadin, Joel Pearson

**Affiliations:** 1 Vanderbilt Vision Research Center and Department of Psychology, Vanderbilt University, Nashville TN, 37240, USA; 2 Department of Brain and Cognitive Sciences and Center for Visual Science, University of Rochester, Rochester, NY, 14627, USA; 3 Department of Ophthalmology, University of Rochester School of Medicine, Rochester, NY, 14642, USA; 4 School of Psychology, The University of New South Wales, Sydney, 2052, Australia

## Abstract

Visual phenomena demonstrating striking perceptual disappearances of salient stimuli have fascinated researchers because of their utility in identifying neural processes that underlie subjective visibility and invisibility. Motion-induced blindness (MIB) is appealing for such purposes because it, like a class of ostensibly related paradigms such as binocular rivalry, features periods of unequivocal subjective disappearances despite constant physical stimulation. It remains unclear, however, exactly how the mechanisms that cause MIB are related to subjectively observed fluctuations in visual awareness. To address this question, we used continuous flash suppression (CFS) to present the MIB stimulus outside visual awareness. Results indicated that MIB occasionally reappeared from suppression with its salient yellow target absent. To quantify this observation, we measured reaction times (RTs) to detect the yellow dot target following visible or perceptually suppressed MIB and indeed found no difference in RTs between these conditions. We also provide evidence that MIB fluctuations can occur without attention. In sum, these experiments indicate that MIB fluctuations are effectively changes in stimulus strength, which under typical conditions result in unmistakable subjective disappearances, but are not inherently fluctuations in stimulus visibility. More broadly, these results challenge the assumed privileged link between bistable stimuli and visual awareness.

Visual demonstrations in which constant sensory stimulation leads to fluctuating subjective experience have inspired great interest among psychologists, philosophers and neuroscientists. Paradigms such as motion-induced blindness (MIB)^1^, binocular rivalry^2^ and others, offer the facility of generating multiple phenomenal states from constant sensory input^3^,^4^. For example, the MIB paradigm is defined by intermittent subjective disappearances of salient targets embedded in a moving background (Fig. 1a)^1^. However, we hypothesized that the seemingly inherent relationship between MIB dynamics and fluctuations in visual awareness^5^,^6^,^7^,^8^ may simply be a function of the typical stimulus conditions under which MIB is viewed. Specifically, we conjectured that MIB dynamics derive from neural processes that, under typical MIB viewing conditions, happen to oscillate around subjective visibility, but under non-typical viewing conditions might not (Fig. 1).

We therefore sought to study MIB under conditions where MIB fluctuations were not yoked to subjective perceptual fluctuations—in other words, conditions under which MIB could have effects that were not visible to the observer. To achieve this we used continuous flash suppression (CFS)^9^ to present MIB outside of visual awareness. In considering what may happen to MIB functions under perceptual suppression, we explored two extreme alternatives (Fig. 1b vs c). One possibility is that perceptual suppression entirely disrupts the mechanisms driving MIB, leading to effective cessation of MIB dynamics during the period of perceptual suppression (Fig. 1b). This alternative indicates that MIB fluctuations are inherently fluctuations in stimulus visibility (visible vs. invisible), which cease once the entire stimulus is made invisible via CFS. On the other extreme (Fig. 1c), CFS has no impact on MIB dynamics, resulting in identical dynamics during visible and invisible presentations of MIB. In this case, MIB fluctuations can be understood as fluctuations in stimulus strength, which under some conditions result in visibility fluctuations. Intermediate effects of CFS, such as a reduction in the magnitude of MIB-induced fluctuations, are also possible.

To differentiate between scenarios outlined in Fig. 1, we asked observers to detect the target item following ‘visible’ (Fig. 2a) or ‘suppressed’ (Fig. 2b) periods of MIB. This allowed us to infer whether MIB dynamics continue or halt during the time period of interest (i.e., CFS or typical viewing). If, at the extreme, MIB is entirely disrupted by CFS, we should expect an elimination of mechanistic fluctuations underlying MIB, resulting in immediate visibility of the target following the cessation of CFS (Fig. 1b). However, if MIB continues to influence the underlying representation of the target even during perceptual suppression, the target should *sometimes* be perceptually suppressed at the reappearance of the MIB stimulus (Fig. 1c).

Preliminary tests revealed that upon the removal of the CFS stimulus, the blue crosses of the MIB stimulus always became visible, but occasionally *without* the salient yellow target dot. To quantify this revealing informal observation, we implemented a simple reaction time task. In brief, observers pressed a key to indicate when, after the removal of the CFS stimulus, they first saw the yellow target dot (Fig. 2b). The hypothesis was that if MIB continues under perceptual suppression, we should see longer reaction times to the target dot (Fig. 1c). Finally, to test the generalizability of our main finding, we also investigated MIB under conditions of inattention^10^ (Fig. 2c,d).

## Method

### Participants

Each observer provided informed consent prior to participating, and all procedures were conducted according to a proposal approved by the University of New South Wales Human Research Ethics Committee (HREC). 8 observers (3 female) participated in the main experiment. 2 observers were authors (KD and JP); the other 6 were naïve to the purpose of the experiment. Our aim was to recruit and test a sample size that matches typical MIB studies, where sample sizes range between 4 and 10 subjects^1^,^11^,^12^,^13^,^14^.

### Apparatus and Procedure

Observers were seated on a height adjustable chair at a distance of 57 cm from a 20” diagonally SONY Multiscan G520 CRT monitor, resolution 1024 × 768 and a refresh rate of 100 Hz. Observers’ heads were stabilized by a chin-rest as they viewed stimuli through a mirror stereoscope. Stimuli were presented using Psychtoolbox Version 3 for MATLAB on a Macintosh MacPro machine.

Each trial consisted of two main segments. First, during an initial period lasting 3 or 5 seconds (randomly determined on each trial), observers were instructed to simply view stimuli. In brief, stimuli (described below) consisted of an MIB stimulus presented to one eye, while the other eye viewed one of the following (depending on trial type; Fig. 2): a fusible fixation circle (MIB), a CFS stimulus (CFS), an RSVP stream (RSVP), or both a CFS and RSVP stimulus (CFS+RSVP). At the end of this initial period, an audible beep was played coincident with the offset of the manipulation (if any), and observers were instructed hit the space bar as soon as they saw the yellow dot (monocular MIB continued until response). On perceptual suppression trials (CFS and CFS+RSVP), observers were instructed to discard any trial on which any part of the MIB stimulus (yellow dot or rotating crosses) broke through suppression prior to the auditory beep. In total, observers discarded a median of 5 (5.9%, discarded trials were reshuffled into the remaining trials) suppression trials per session, indicating that suppression was generally strong throughout the experiment. Observers also discarded 100% of CFS catch trials (described below).

The MIB stimulus consisted of a 6 × 6 degree square containing small blue crosses (8 × 8) rotating at 3 degrees/second (direction randomly selected on each trial). When the 16 arcmin diameter yellow dot target was present (see below), it was centered 2 degrees above or below fixation (randomly selected on each trial), and was centered within a 32 arcmin diameter black buffer (to eliminate transients when the crosses disappeared behind the target^1^). On MIB only trials (Fig. 2a), these MIB stimuli were presented to one eye while the other eye viewed a black screen with a fusible central fixation point. On CFS trials (Fig. 2b), one eye was presented with the MIB stimulus, while the other eye viewed a dynamic Mondrian pattern consisting of 75 colored rectangles that were redrawn every 100 ms^9^. After the response cue, MIB continued in the eye in which it was originally presented until the observer reported seeing the target.

In the RSVP condition (Fig. 2c), rapidly changing letters and characters were presented at fixation, and observers were instructed to count the number of “=” and “#” targets that appeared. On CFS+RSVP trials (Fig. 2d), this RSVP stream was presented on top of the CFS stimulus (described above). Participants’ responses were entered at the end of the trial, after the report of the yellow dot target (each trial contained 0–2 RSVP targets). Targets never occurred within 500 ms of the start or end of the manipulation period, and a second target could not occur less than 500 ms after the first. This task was challenging but completed with good accuracy (mean 83.0% for RSVP trials, 85.9% for CFS+RSVP trials, with no difference in accuracy between the two t_7_ = 1.2, p = 0.27).

Observers completed the main experimental trials in 2 sessions. In one session, observers completed the MIB only and CFS trials (data shown in Fig. 3); in the other session, observers completed the RSVP and CFS+RSVP trials. Session order was counterbalanced across observers. These condition pairings were used to minimize task switching across trials during the experiment and to make sure that the data for the critical comparison (CFS vs. MIB only) was collected in the same session. Each session consisted of 170 trials: 160 experimental trials and 10 catch trials, on which CFS covered only half of the MIB stimulus (observers correctly discarded 100% of catch trials, 10 per session).

On each trial, the yellow dot was presented in one of 3 ways. In the main test condition, the yellow dot was present throughout the entirety of the trial. In the main control condition (OFF/ON), the yellow dot was physically absent during the initial 3 or 5 seconds, and was turned on coincidentally with the auditory beep. In a second control condition (data not reported), the dot was physically on during the initial 3- or 5-second segment, but was turned off coincidentally with the auditory beep. After 1 second, the dot was turned back on. These trials served primarily to prevent participants from immediately hitting the space bar upon hearing the beep, and results in this condition were not of particular theoretical interest (observers successfully waited for the appearance of the yellow dot target, as evidenced by slow RTs in this condition).

Observers self initiated each trial, and were informed prior to each trial about the upcoming trial type. Additionally, observers completed a short practice block prior to beginning each experimental session. During the first practice, the luminance of the crosses and yellow dot were adjusted to one of three values used for each individual to ensure strong suppression and effective MIB. For each observer, identical luminance values were used on all trials and in all sessions of the experiment.

We performed an analysis to estimate the proportion of trials on which the target was perceptually invisible at the start of the response period (Figs 3d and 4d). “Disappearance trials” were defined as those Test trials with RTs longer than 95% of RTs in the corresponding OFF/ON control. This is akin to asking which trials in the Test condition would be considered outliers in the OFF/ON control.

**Figure 1:**
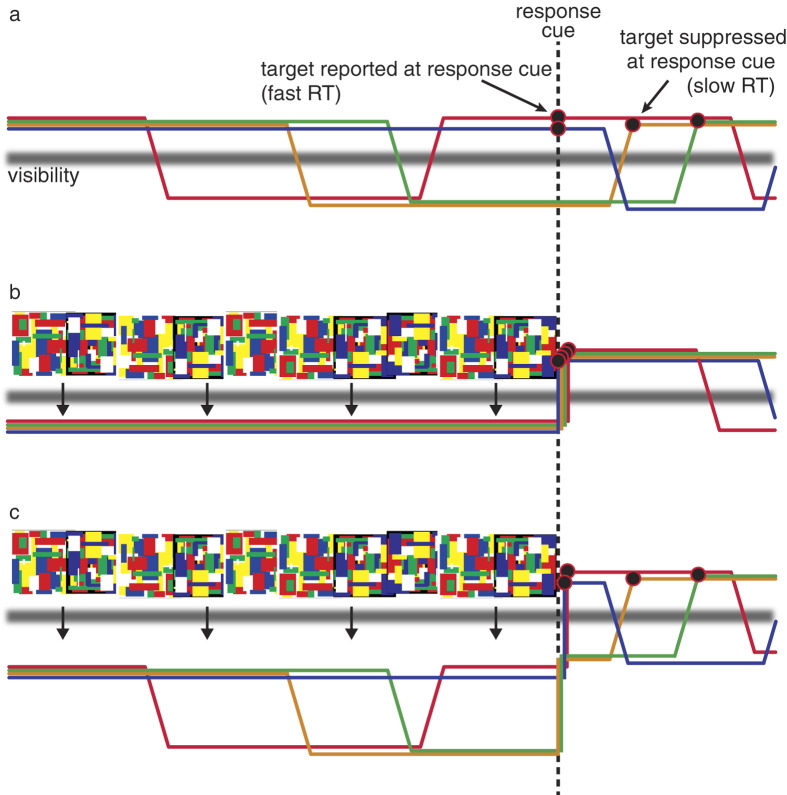
Models of MIB dynamics under typical overt viewing and perceptual suppression conditions. (**a**) In typical MIB, fluctuations in the target representation around subjective visibility (shown by a gray line in all panels) lead to occasional perceptual disappearances. For a fixed response cue time, this can result in fast or slow RTs depending the current state of MIB dynamics. (**b**) If MIB fluctuations stop under perceptual suppression, bringing the MIB stimulus back into visibility should simply result in the onset of MIB dynamics (i.e., same dynamics as the beginning of panel **a**), and thus, consistently fast RTs to target onset. (**c**) Instead, we find variable RTs following CFS of MIB, suggesting that MIB fluctuations continue even under suppression. Note that the “visibility” line in Fig. 1 is not intended to imply a hard threshold, and is meant only to visually separate states of target detection and perceived target absence. Moreover, distinctly binary percepts (“target present” or “target absent”) could arise either from gradual (as depicted) or step-like fluctuations in stimulus strength (e.g., during binocular rivalry, alternating perceptual states are experienced even though stimulus representations are continuously changing in strength over time^26^,^27^). The outlined logic holds regardless of whether stimulus fluctuations are gradual or abrupt.

We also used post-hoc Bayes factor analyses to further examine our data. Such an analysis allows one to quantify the evidence in favor of or against a null effect, with the resulting value indicating odds in favor or against the null effect^15^. These calculations were performed using the Bayes factor calculator developed by Rouder and colleagues, available at: http://pcl.missouri.edu/bayesfactor. For both tests, the null hypothesis is an effect size of 0, while the alternative hypothesis is specified as a Cauchy distribution on effect size. For both hypotheses, the Jeffreys prior on variance is used. These details are as recommended for default Bayes factor analyses as they involve few assumptions^15^. In the first analysis, we compared the Test condition results under each manipulation to those in the MIB Only condition (akin to post-hoc paired t-tests of the CFS, RSVP, and CFS+RSVP Test trials vs. MIB Only). Here, the null hypothesis means that MIB continues unaltered under CFS, RSVP, or both (e.g. Fig. 1c). In the second analysis, we compared Test conditions to OFF/ON conditions under each manipulation (akin to post-hoc paired t-tests of dark vs. light bars, Figs 3 and 4). Here, the null hypothesis of 0 effect size is illustrated in Fig. 1b.

To ensure that long RTs in the CFS condition were in fact due to MIB and not caused by direct suppression of the target element by the CFS stimulus, we conducted a separate control experiment (n = 8, 3 also participated in main experiment) in which CFS was used to suppress an MIB stimulus that had either rotating (i.e., conventional MIB) or stationary cross elements (i.e., a single static frame from the MIB animation). On stationary trials, the MIB frame remained static after the response cue to avoid the introduction of a motion transient. We also ran a separate version of the stationary condition with those same 8 observers where motion of the MIB stimulus began coincident with the response cue (CFS offset), to perceptually match the response period to the main experiment. The rationale behind manipulating motion of the MIB stimulus was that if CFS were directly responsible for MIB target disappearances, then we should see evidence for those disappearances with both rotating and static MIB. If motion remains crucial to this effect (as with typical MIB), then RTs on rotating trials should be longer. On these trials, the response cue always occurred 5 s after trial onset. Observers discarded a median of 18.1% (discarded trials were repeated) trials due to suppression breaks across experiments, and correctly discarded 100% of catch trials (5 per session).

## Results

We first directly compared MIB under typical overt viewing and perceptual suppression conditions (Fig. 3). MIB alone yielded slow RTs (mean ~ 900 ms; Fig. 3b, red bars). These slow RTs are a direct consequence of occasional perceptual disappearances of the yellow dot target caused by the rotating blue pattern—the key defining feature of MIB. Crucially, suppressing MIB yielded similarly slow RTs (planned paired t-test; t_7_ = 0.26, p = 0.8; Fig. 3a,b), indicating that observers had to occasionally wait for the target to reappear under both viewing conditions. Furthermore, a Bayes factor analysis^15^ indicates that a lack of difference between CFS and MIB (Fig. 1c) is 3 times more likely than a hypothesis postulating a real difference between the two conditions (see Method).

To confirm that observed slow RTs were caused by MIB suppression of the target stimulus, the test trials were intermixed with OFF/ON control trials in which the target was *physically* absent prior to the response cue (OFF) and shown only after manipulation offset (ON). Under this condition, no MIB induced disappearances are expected, resulting in faster RTs. Indeed, we found a significant effect of trial type (F_1,7_ = 16.3, p = 0.005), but no main effect of awareness nor interaction (F_1,7_ = 0.54, 1.8; p = 0.49, 0.22, Fig. 3b). Furthermore, each individual observer showed slower RTs on Test than OFF/ON control trials (Fig. 3a, small dots) for both MIB and CFS conditions. Likewise, a direct comparison of RT distributions revealed that short RTs were less likely, and long RTs more likely, during Test than OFF/ON control trials in both awareness conditions (Fig. 3c). We also estimated the proportion of trials featuring target disappearances (see Methods) and found no effect of CFS (Fig. 3d; t_7_ = 0.85, p = 0.42). A second Bayes factor analysis comparing Test to OFF/ON trials in the CFS condition indicated that it is 4 times more likely that there is a real difference between two trial types than not, further suggesting that MIB does not stop under CFS (as in Fig. 1c).

One possible explanation of our results is that it was CFS, and not MIB, that caused the target to occasionally disappear. Under this scenario, perceptual disappearances are induced by direct suppression of the target dot by the CFS stimulus, meaning that rotation of the MIB stimulus is not crucial to the effect. To test this alternative hypothesis, we presented CFS to one eye and either typical, rotating MIB^11^ or a static MIB frame to the other eye. Comparing these results to the main experiment replicated our key finding of longer RTs on trials when rotating MIB was suppressed as compared to stationary control trials (F_1,14_ = 13.62, p = 0.002) with no main effect of experiment (F_1,14_ = 0.01, p = 0.94) and no interaction between experiment and trial type (F_1,14_ = 0.12, p = 0.73). Direct comparison of suppressed static vs. rotating MIB suggested, as expected, longer RTs on rotating trials (t_7_ = −2.29, one-tailed p = 0.028), with RTs for static MIB comparable to the OFF/ON control trials (OFF/ON = 690ms; stationary MIB = 699 ms) and rotating MIB trials replicating the main experiment (905 ms and 877 ms, respectively). We also ran an additional stationary control where the stationary MIB condition was modified so that MIB started moving after CFS offset. This control condition was designed to perceptually match the main CFS condition. Results were statistically indistinguishable from original stationary control (708ms; t7 = 0.09, p = 0.93). Taken together, these results indicate that longer RTs in the CFS condition were likely driven by ongoing MIB dynamics (Fig. 1c). In addition, stationary MIB conditions demonstrate that faster RTs in the OFF/ON conditions were not simply due to facilitated detection of the target dot (due to its transient onset).

**Figure 2:**
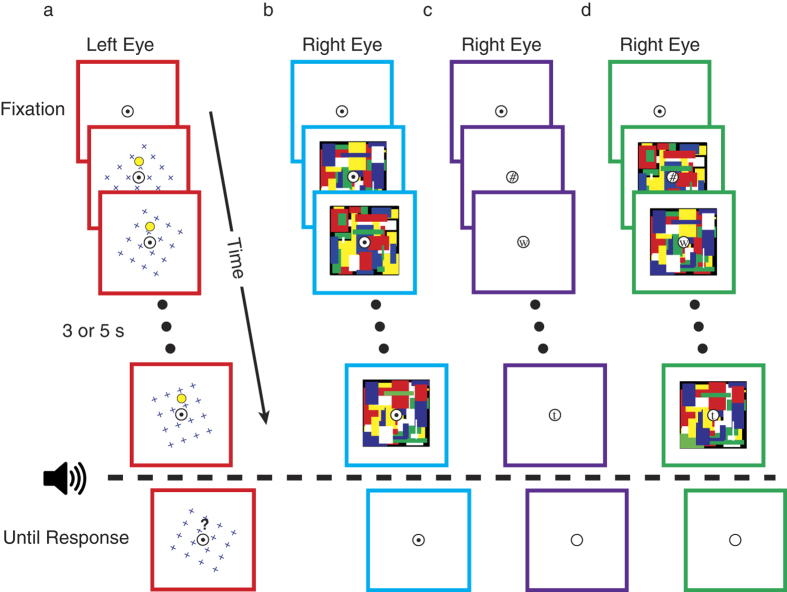
Method. (**a**) On all trial types, one eye (here, left) viewed an MIB stimulus consisting of rotating blue crosses and a yellow dot target. During MIB Only trials (red), the other eye viewed only a central fixation point. (**b**) On “CFS” trials (blue), a dynamic Mondrian pattern (10 Hz) was presented to the other eye, resulting in complete perceptual dominance of the Modrian pattern until its removal. (**c**) On “RSVP” trials (purple), observers attended to a rapidly changing stream of letters at fixation. (**d**) On “CFS + RSVP” trials (green), both manipulations (**b** & **c**) were used in concert. Note: colored borders were not presented and serve to facilitate matching to data in Figs 3 and 4.

**Figure 3:**
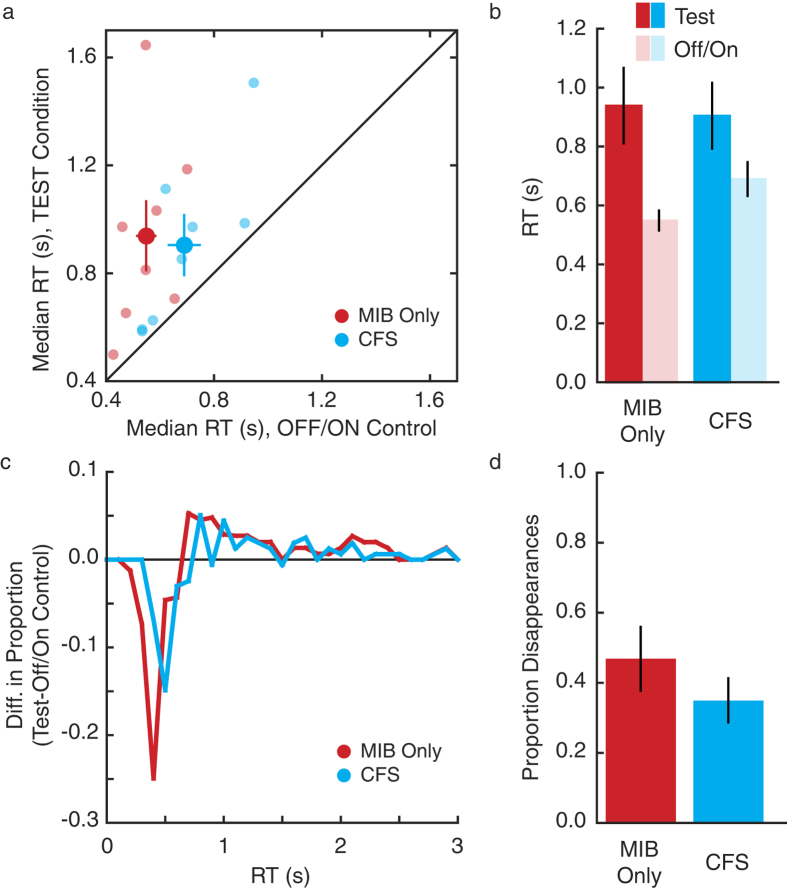
Results from perceptual suppression. (**a**,**b**) RTs to detect the target item were unaffected (large dots in **a**, dark bars in **b**) when MIB was perceptually suppressed by CFS. On OFF/ON control trials, RTs were faster for both conditions and all observers individually (small dots in **a**, light bars in **b**). (**c**) The difference between RT histograms (100ms bins) for Test and OFF/ON control trials shows that fast RTs were less likely, and long RTs were more likely, on Test than OFF/ON control trials. (**d**) The proportion of target disappearances in two conditions was unaffected. Bars in (**b** & **d**) and large dots in **a** indicate group averages; small dots in **a** indicate individual medians (n = 8). Error bars indicate SEM.

Motivated by evidence that MIB transpires under perceptual suppression, we tested whether this result generalizes to inattention. Here, we diverted attention from MIB by utilizing a demanding rapid serial visual presentation (RSVP) detection task at fixation (Fig. 2c). Additionally, we used both manipulations in concert to divert attention and perceptually suppress MIB (Fig. 2d).

**Figure 4:**
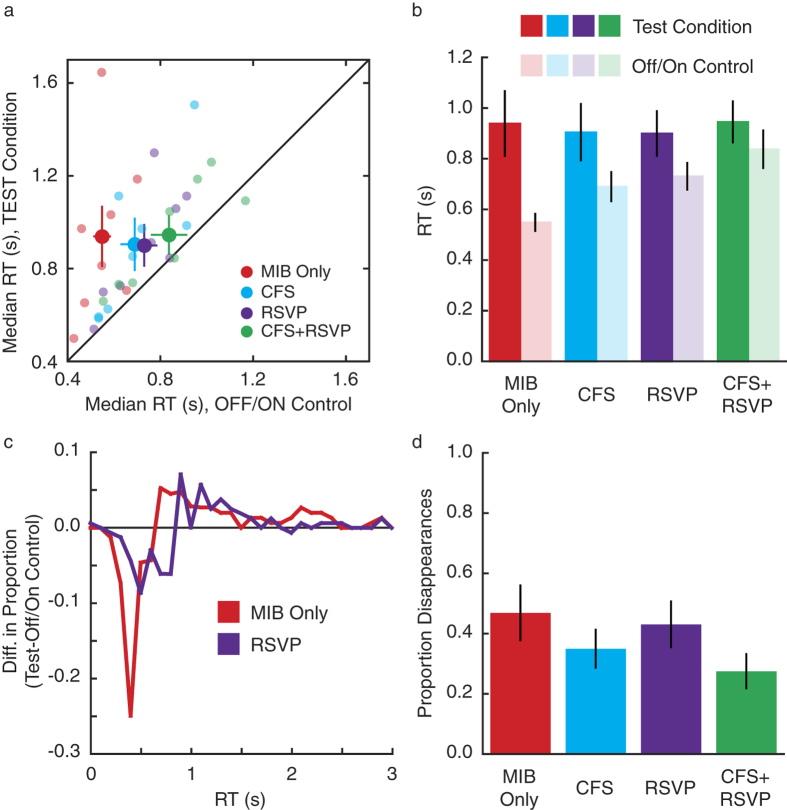
Results from inattention. (**a**,**b**) RTs to detect the target item were unaffected (large dots in **a**, dark bars in **b**) when MIB was perceptually suppressed by CFS, unattended (RSVP), or both (CFS+RSVP). On OFF/ON control trials, RTs were faster for all conditions and most observers individually (small dots in **a**, light bars in **b**). (**c**) The difference between RT histograms for Test and OFF/ON control trials shows that fast RTs were less likely, and long RTs were more likely, on Test than OFF/ON control trials in both the MIB Only and RSVP conditions. (**d**) The proportion of target disappearances in four conditions was unaffected by manipulation. Bars in (**b** & **d**) and large dots in **a** indicate group averages; small dots in **a** indicate individual medians (n = 8). Error bars indicate SEM. Data for MIB Only and CFS conditions is the same as Fig. 3.

Comparing results from inattention and suppression manipulations indicated that average RTs to detect the target item were unaffected under all conditions (F_3,21_ = 0.08, p = 0.97; Fig. 4a,b). Bayes factors comparing Test trials for each condition (CFS, RSVP and CFS+RSVP) to those in the MIB Only condition indicated that no difference between conditions was 3 times more likely (for each comparison) than a real difference between conditions. In addition, comparison of RTs in Test vs. OFF/ON control trials indicated a main effect of trial type (OFF/ON vs. Test, F_1,7_ = 22.37, p = 0.002), but no main effect of manipulation condition (F_3,21_ = 1.54, p = 0.23; Fig. 4b). There was a marginal interaction (F_3,21_ = 2.78, p = 0.07), resulting from longer RTs on OFF/ON control trials in the manipulation conditions, especially when CFS and RSVP were combined. This is likely due to task-switching costs^16^ induced by the dual task nature of the CFS+RSVP condition (these would be chiefly reflected in OFF/ON control trials). Individual data indicated that nearly all observers under all manipulations showed slower RTs to test than OFF/ON control trials (Fig. 4a). As with the perceptual suppression condition, we found that short RTs were less likely, and long RTs more likely, for Test than OFF/ON control trials in the RSVP condition (Fig. 4c), and that the proportion of perceptual disappearances was unaffected by manipulation (F_3,21_ = 1.31, p = 0.3; Fig. 4d). Bayes factors for Test vs. OFF/ON trials in each manipulation suggested that it was 5 (MIB Only), 4 (CFS), 4 (RSVP), and 3 (CFS+RSVP) times more likely that there is a real difference between trial types than not, supporting the argument that MIB does not stop outside awareness or attention. Thus, we find strong evidence that MIB continues under conditions of both perceptual suppression and inattention when tested separately. We also find evidence that MIB transpires when both manipulations are used together, although this strength of the finding is likely attenuated by high task-switching costs in this condition.

## Discussion

Our main findings indicate that critical dynamics underlying MIB continue when MIB is perceptually suppressed. In other words, target fluctuations that are witnessed overtly during typical MIB viewing are likely driven by mechanisms independent from, or operating prior to, neural mechanisms underlying perceptual suppression in CFS. This result supports the hypothesis that MIB fluctuations are essentially fluctuations in stimulus strength, which under some conditions result in visibility fluctuations but are not inherently visibility fluctuations. Stimulus fluctuations during MIB could arise early in visual processing^7^,^17^ and/or from fundamental visual processes such as adaptation^12^ and motion streak suppression^13^. A strong suppressor (CFS) does not halt these fluctuations; instead it simply makes their immediate perceptual consequences invisible (Fig. 1c).

We also find that the independence of MIB from perceptual suppression generalizes to inattention. Attention and awareness are highly intertwined concepts, and the exact relationship between these processes has been the subject of considerable debate^18^. In our results, we find that the dynamics of MIB are similarly unaffected by suppression or inattention. Intriguingly, this pattern highlights a difference between MIB and binocular rivalry, which was recently found to stop outside of attention^19^,^20^. This dissociation may help elucidate mechanisms giving rise to perceptual disappearances under various forms of visual bistability^21^, and is consistent with recent evidence for a basic role of attention in binocular rivalry^20^.

Evidently, finding neural fluctuations matched to the perceptual dynamics of MIB^7^,^8^, and by extension, other related paradigms^5^,^6^, does not necessarily imply that one has isolated the neural bases of visual awareness^22^,^23^. Instead, one is perhaps likely to find prerequisite processes that are critically involved in the specific paradigm in use^24^,^25^. Thus, future work toward elucidating the exact combination of neural processes that cause MIB dynamics is critical.

## Additional Information

**How to cite this article**: Dieter, K. C. *et al.* Motion-induced blindness continues outside visual awareness and without attention. *Sci. Rep.* **5**, 11841; doi: 10.1038/srep11841 (2015).
